# Supplementary material for: The microbiota of Amblyomma americanum reflects known westward expansion
Source: PLoS One. 2024 Jun 10;19(6):e0304959. doi: 10.1371/journal.pone.0304959 (PMC11164389; doi:10.1371/journal.pone.0304959)
Supplement: S4 File — (DOCX) [file pone.0304959.s004.docx]

**S4 File.** Real-time PCR primers and probes used to investigate presence of medically important bacteria in *Amblyomma americanum* [collected from sites within their Historic or expanded range in the USA]. All primers and probes provided in the 5’ to 3’ direction.

| **Pathogen** | **Target** | **Specifications and parameters** | **Reference number** |
| --- | --- | --- | --- |
| *Ehrlichia* *chaffeensis* | *dsb* | Primer F: TTGCAAAATGATGTCTGAAGATATGAAACA  Primer R: GCTGCTCCACCAATAAATGTATCYCCTA  Probe: VIC-TGCTAGTGCTGCTTGAACAGCTTTCAGTGAT-QSY | **100** |
| *Ehrlichia* *ewingii* | *dsb* | Primer F: TTGCAAAATGATGTCTGAAGATATGAAACA  Primer R: GCAGCTCCACCAATGAATGTATTTCCAA  Probe: 6FAM-AGCCAATGCTGCACGTACTGCTTTCAATGAT-QSY | **100** |
| *Anaplasma phagocytophilum* | *msp*2 | Primer F: AGTTTGACTGGAACACACCTGATC  Primer R: CTCGTAACCAATCTCAAGCTCAAC  Probe: 6FAM-TTAAGGACAACATGCTTGTAGCTATGGAAGGCA-QSY | **101** |
| *Rickettsia rickettsii* | hypothetical protein A1G_04230 | Primer F: AAATCAACGGAAGAGCAAAAC  Primer R: CCCTCCACTACCTGCATCAT  Probe: 6FAM- TCCTCTCCAATCAGCGATTC -QSY | **102** |
