# Supplementary material for: The microbiota of Amblyomma americanum reflects known westward expansion
Source: PLoS One. 2024 Jun 10;19(6):e0304959. doi: 10.1371/journal.pone.0304959 (PMC11164389; doi:10.1371/journal.pone.0304959)
Supplement: Attachment — Submitted filename: Author responce Amblyomma americanum.docx [file pone.0304959.s008.docx]

Overall, the manucript titled “The microbiota of *Amblyomma americanum* reflects known westward expansion” is an interesting study describing microbiota of *Amblyomma americanum*  in some region of united states.

The manuscript should be revised by the authors in order to be published. I addressed some point below line by line.

The manuscript is definitely too long, thus I suggest shortening it by cutting some parts on the too detailed paragraphs ([best of speech is the short and](https://context.reverso.net/translation/english-arabic/best+of+speech+is+the+short+and) informative one (.

**Title**

The title is ambiguous and needs more clarification. What does the word (westward expansion) refer to?

**Abstract**

The abstract must involve the tick borne pathogens that determined by researchers

**Introduction**

Line 55: it is preferred to replace “driver” with -----motivation

Line 56: geographic range expansion---to-----geographic distribution

Line 73: You could have mentioned briefly the functional roles of the tick microbiota

**Materials and Methods**

Line 117: 189 *A. americanum* specimens collected at 24 localities, this number is too small. You can describe your limitation and difficulties to obtain more samples.

Line 148: Mention the active ingredient of bleach in parentheses.

Line 146: you are mentioned “we extracted genomic DNA from each individual tick” , This contradicts your statement “Ticks, randomly chosen, were surface”, so you must replace the second sentence with “all ticks sterilized with ……

Line 152: ATL----- Write the full name and abbreviation in parentheses

Line 157-176; line 293-303: I suggest making small table including gene, Primer type, Primer sequence, PCR product size (base pair), thermocycling conditions, References for each primer

Line 177: Bioinformatic analyses--- Authors should rephrase this section so that it is clear and concise

**Results**

Line 323, 324: must be written in discussion

Fig 2: it is preferred to be changed with good clear colors and resolution, so that the readers can recognize the details of the figure easily.

Fig. 3: the meaning of the abbreviated letters must be written on the figure as fig.4

Line 364: please write abbreviation in parentheses as example (W- region) or any other format to avoid the confusion with other words ---within whole manuscript

**Discussion**

Line 541; structuring—to ---structure

Line 587, 587; reformulate the sentence
