# Supplementary material for: The microbiota of Amblyomma americanum reflects known westward expansion
Source: PLoS One. 2024 Jun 10;19(6):e0304959. doi: 10.1371/journal.pone.0304959 (PMC11164389; doi:10.1371/journal.pone.0304959)
Supplement: Attachment — Submitted filename: Response to reviewers PONE_013124.docx [file pone.0304959.s009.docx]

**Rebuttal letter for the [PONE-D-23-34044] Manuscript**:

“The microbiota of *Amblyomma americanum* reflects known westward expansion”

**Reviewer 1**

We appreciate your suggested corrections and have incorporated them to the text.

**Reviewer 2**

Overall, the manucript titled “The microbiota of *Amblyomma americanum* reflects known westward expansion” is an interesting study describing microbiota of *Amblyomma americanum*  in some region of united states.

The manuscript should be revised by the authors in order to be published. I addressed some point below line by line.

The manuscript is definitely too long, thus I suggest shortening it by cutting some parts on the too detailed paragraphs ([best of speech is the short and](https://context.reverso.net/translation/english-arabic/best+of+speech+is+the+short+and) informative one (.

We thank you for your helpful comments. We agree the manuscript is quite lengthy. Overall, we have been as selective and direct with our language as possible, and believe the length mostly reflects the depth of the analysis we performed and the high-quality discussion we provide. Nonetheless, we agree it can be tightened, and we have edited it in places to reduce length.

**Title**

The title is ambiguous and needs more clarification. What does the word (westward expansion) refer to?

The term westward can be used as an adjective or adverb of directionality. We chose it since it is a term frequently used when referring to the movement of populations expanding their geographic range. The term expansion is a standard term in the ecological literature but can also be understood through its standard definition “the action of becoming larger or more extensive.” The term “westward” coupled with the term “expansion” connotes that the tick population has become larger in a westerly direction. For instance, refer to the following Plos One publication where the authors are referring to the *A. Americanum* expansion across the United States.

<https://journals.plos.org/plosone/article?id=10.1371/journal.pone.0209082>

“Further northward and westward expansion of these ticks can be expected as a result of ongoing climate change, under both low- and high-emissions scenarios.”

We respectfully request keeping our original title, as we believe it succinctly and directly communicates the main findings of the paper.

**Abstract**

The abstract must involve the tick borne pathogens that determined by researchers

As suggested by the reviewer we have made mention of this component of our research in the abstract in lines 46-48 reading as follows: “We also found that prevalence of *Ehrlichia chaffeensis*, *E. ewingii*, and *Anaplasma phagocytophilum* ranged from 3.40 – 5.11% and did not significantly differ by region. *Rickettsia rickettsii* was absent from our samples.”

**Introduction**

Line 55: it is preferred to replace “driver” with -----motivation

In this particular sentence the word “driver” is specifically referring to a hypothetical cause for the increase in tick borne disease cases. We believe the word “driver” fits in the epidemiologic context but we changed it to “cause” in line 57 to use more conventional vocabulary.

Line 56: geographic range expansion---to-----geographic distribution

In this particular case we ask the reviewer to consider that the phenomenon of “range expansion” is distinct from geographic distribution and well described for this species as other species in the literature. The purpose of this paper is in fact to propose microbiota data as an additional layer of information to understand the active phenomenon of range expansion.

Line 73: You could have mentioned briefly the functional roles of the tick microbiota

The reviewer is correct. We had detailed these functions in previous versions of the manuscript but for the purpose of keeping the introduction concise we focused the contents of this section to the main theme of the paper (range expansion) and how microbiomes have been studied in this context. Nonetheless, we do provide references to the reader to access information regarding the functional roles of microbial communities in their tick hosts. We believe this is sufficient to provide context to the reader, while mentioning some of the functional roles of tick microbiomes in the discussion.

**Materials and Methods**

Line 117: 189 *A. americanum* specimens collected at 24 localities, this number is too small. You can describe your limitation and difficulties to obtain more samples.

The reviewer is correct when noticing that the number of collected individuals can be considered small when compared to studies performed with laboratory vector colonies. The sample collections in this study were performed by a small group of individuals aiming to cover multiple states within the historic and expanded range. Recovering a fairly small number of individuals with the cloth dragging method, or other methods in fact, is not uncommon, but rather expected. As example we provide here a list of studies cited in our manuscript with a range of samples between 39 to 998. Some of them were made in longer collection periods in the wild (e.g. 3 years) or with different methods. One refers to samples collected by veterinary doctors at a clinic (Reference #114).

There are indeed multiple limitations and difficulties to obtain more samples. But given that our sample number is well within the numbers of previously published studies, some specifically addressing *A. americanum*, range expansion or microbiota, we consider the study successful and believe it is not necessary to describe the limitations in the manuscript. Nonetheless, we do highlight the relevance of a balanced sample size to better understand the ecological process quantification analysis in the context of expansion as did reference #57.

| **Number of ticks** | **Reference #** |
| --- | --- |
| 998 | 119 |
| 145 | 114 |
| 131 | 109 |
| 120 | 111 |
| 92 | 57 |
| 90 | 113 |
| 89 | 110 |
| 50 | 31 |
| 39 | 118 |

Line 148: Mention the active ingredient of bleach in parentheses.

We have added the active bleach used at this step as suggested by the reviewer. The sentence now reads: “…commercial bleach (Clorox, NaOCl active bleach)

Line 146: you are mentioned “we extracted genomic DNA from each individual tick”, This contradicts your statement “Ticks, randomly chosen, were surface”, so you must replace the second sentence with “all ticks sterilized with ……

We have re written the sentence to convey the idea better. In fact, all samples were sterilized. The “randomly chosen” refers to the fact each of them was chosen in random order. The sentence now reads “All the ticks, in random order, were surface sterilized…”

Line 152: ATL----- Write the full name and abbreviation in parentheses

This buffer is a proprietary reagent from Qiagen and each of the bottles are named with abbreviations. See for instance: <https://www.qiagen.com/us/products/discovery-and-translational-research/lab-essentials/buffers-reagents/buffer-atl> Readers acquainted or new to the extraction kit would find ATL as sufficient reference or information to follow the protocol suggested by Qiagen. We would appreciate if the reviewer considers this in order to keep that line as it stands.

Line 157-176; line 293-303: I suggest making small table including gene, Primer type, Primer sequence, PCR product size (base pair), thermocycling conditions, References for each primer

We appreciate the suggestion from the reviewer. In the particular case of the high throughput sequencing (HTS) of regions V4-V5 we consider that the information in lines 157-176 contains all the relevant parameters, aspects and references a reader may require. Furthermore, HTS was performed at the Argonne National Laboratory and we do not possess the specific information regarding thermal cycling. As for the pathogen detections (lines 293-303), we can provide all this information in a supplementary table. We believe this will also help reduce the length of the manuscript. For real-time PCR product size is not relevant because all RT-PCR targets are held to a small fragment usually 120-150bp and visualized via fluorescent curve rather than a gel so it is not common practice to include product size in tables or text in the literature. For the rest of the information see the table below. It will be included as S4 File in the manuscript.

**S4 File.** Real-time PCR primers and probes used to investigate presence of medically important bacteria in *Amblyomma americanum* [collected from sites within their Historic or expanded range in the USA]. All primers and probes provided in the 5’ to 3’ direction.

| **Pathogen** | **Target** | **Specifications and parameters** | **Reference number** |
| --- | --- | --- | --- |
| *Ehrlichia* *chaffeensis* | *dsb* | Primer F: TTGCAAAATGATGTCTGAAGATATGAAACA  Primer R: GCTGCTCCACCAATAAATGTATCYCCTA  Probe: VIC-TGCTAGTGCTGCTTGAACAGCTTTCAGTGAT-QSY | **100** |
| *Ehrlichia* *ewingii* | *dsb* | Primer F: TTGCAAAATGATGTCTGAAGATATGAAACA  Primer R: GCAGCTCCACCAATGAATGTATTTCCAA  Probe: 6FAM-AGCCAATGCTGCACGTACTGCTTTCAATGAT-QSY | **100** |
| *Anaplasma phagocytophilum* | *msp*2 | Primer F: AGTTTGACTGGAACACACCTGATC  Primer R: CTCGTAACCAATCTCAAGCTCAAC  Probe: 6FAM-TTAAGGACAACATGCTTGTAGCTATGGAAGGCA-QSY | **101** |
| *Rickettsia rickettsii* | hypothetical protein A1G_04230 | Primer F: AAATCAACGGAAGAGCAAAAC  Primer R: CCCTCCACTACCTGCATCAT  Probe: 6FAM- TCCTCTCCAATCAGCGATTC -QSY | **102** |

Line 177: Bioinformatic analyses--- Authors should rephrase this section so that it is clear and concise.

We have modified the section for clarity.

**Results**

Line 323, 324: must be written in discussion

We kindly request leaving this short section unchanged. We do discuss the first sentence briefly again in the discussion, and as the finding of a 3^rd^ dominant member in our data set was limited to only 3 samples, the description in the results and its brief discussion seem to be adequate for this level of observation, especially when aiming to keep the manuscript short. In the Discussion section, lines 511-517 in the original manuscript, we approach these findings in a manner that encompasses both the dominance of a taxa and the variability of other dominant taxa reports for this species. Furthermore, we point the reader to a reference where the factor of surface sterilization influencing the membership of tick associated microbiota is discussed.

Fig 2: it is preferred to be changed with good clear colors and resolution, so that the readers can recognize the details of the figure easily.

For this figure we used a data visualization tool to provide us with a contrasting palette that was both clear and color blind friendly [https://medialab.github.io/iwanthue/]. As it stands, we consider that the figure conveys effectively an overview of the community composition to the extent to which we used this to describe the microbiota. Nonetheless we acknowledge the observation from the reviewer when it comes to the figure in the PDF. In fact the original figure has a better resolution, but it was reduced when we processed with the PACE tool as PLOS ONE requires. The figure uploaded to the system, and to which the reviewer should have had access, is 9.12MB and between 300-600dpi of resolution. Both parameters are in line with the instructions to authors. We will ensure that the quality of the figure is improved in the final formatting and version of the manuscript.

Fig. 3: the meaning of the abbreviated letters must be written on the figure as fig.4

The figure legend has been modified to address the issue. Both Figure 3 and 4 are plotted with abbreviations to minimize cluttering them with text.

Line 364: please write abbreviation in parentheses as example (W- region) or any other format to avoid the confusion with other words ---within whole manuscript

As the reviewer noted, the manuscript is already quite lengthy. Among the decisions we took aiming to reduce the words and/or characters was to define the abbreviation for each of the regions within the Materials and Methods section. As the reviewer can read in lines 122 to 127 in the original manuscript, we provide the meaning of the abbreviations there, and stress to the reader we will refer to the regions with them throughout the manuscript. We believe that spelling out each region would add unnecessary verbosity to the manuscript. .

**Discussion**

Line 541; structuring—to ---structure

The word has been changed to “structure” as suggested.

Line 587, 587; reformulate the sentence

The sentence has been reformulated to read as follows: “In ticks, bacterial members of the microbiome have been shown to influence locomotion (126) generally increasing tick mobility (37).”
